# Safety Analysis of Extended Platelet Shelf-Life with Large-Volume Delayed Sampling on BACT/ALERT^®^ VIRTUO^®^ in Australia

**DOI:** 10.3390/microorganisms11092346

**Published:** 2023-09-19

**Authors:** Anthea Cheng, Anindita Das, Khin Chaw, Peta M. Dennington, Claire E. Styles, Iain B. Gosbell

**Affiliations:** 1 Donor and Product Safety Policy Unit, Australian Red Cross Lifeblood, West Melbourne, VIC 3003, Australia; 2 Clinical Microbiology, ACT Pathology, Garran, ACT 2606, Australia; 3 Faculty of Health, University of Canberra, Bruce, ACT 2617, Australia; 4 MetroSouth Public Health Unit, Eight Mile Plains, QLD 4113, Australia; 5 Pathology Services, Australian Red Cross Lifeblood, Alexandria, NSW 2015, Australia; 6 School of Medicine, Western Sydney University, Penrith, NSW 2747, Australia

**Keywords:** transfusion-transmitted bacterial infection (TTBI), bacterial contamination screening (BCS), platelet shelf-life, large-volume delayed sampling (LVDS), platelet component

## Abstract

Transfusion-transmitted bacterial infection (TTBI) is the leading cause of transfusion-transmitted infections. Platelet components are more likely to be associated with bacterial contamination due to their storage requirements. Australian Red Cross Lifeblood introduced the bacterial contamination screening (BCS) of all platelet components in 2008. The process was recently updated with the use of BACT/ALERT^®^ VIRTUO^®^, a large-volume delayed sampling (LVDS) protocol and extending platelet shelf-life to seven days. This article describes the results from the routine BCS of platelet components in Australia. Use of VIRTUO has resulted in lower false-positive rates, reducing wastage and improving platelet inventory. Our findings show that the combination of LVDS and VIRTUO improves the safety of platelet transfusions through earlier time to detection, especially for pathogenic bacterial species. Pathogenic bacteria grew within 24 h of incubation with a clear delineation between pathogenic and non-pathogenic species. The data show this protocol is very safe, with no TTBI cases during this time. There were no TTBI reports in recipients of platelet components that subsequently had a positive culture with *Cutibacterium* species, probably due to the low pathogenic potential of these organisms and slow replication in aerobic platelet bags. We conclude there is no advantage in incubating culture bottles beyond five days.

## 1. Introduction

Transfusion-transmitted bacterial infection (TTBI) is the leading cause of transfusion-transmitted infections and in Australia, it is reported to occur in 1:250,000 platelet transfusions and 1:2.5 million red blood cell transfusions [1]. Platelet components are more likely to be associated with bacterial contamination due to their storage at room temperature, which promotes bacterial growth. Improvements and changes in the collection, processing, storage, and management of blood and blood components over the past two decades have substantially reduced the risk of TTBI [2,3].

Bacterial contamination is unavoidable during blood collection and processing, despite strict aseptic techniques due to multiple factors, including occult bacteraemia in the donor, residual bacteria from the skin or skin appendages contaminating the donation despite optimal skin decontamination processes, equipment defects, or non-compliance with the established process. In a recent systematic review, it was estimated that the bacterial contamination rate for platelet components was 1 in 1961 [4]. Automated microbial detection systems for bacterial contamination screening (BCS) are commonly used to decrease the risk of TTBI from a platelet transfusion [5]. Australian Red Cross Lifeblood (Lifeblood) performs BCS on all platelet components as part of mandatory testing, prior to release into inventory. Following inoculation into aerobic and anaerobic culture bottles and loading them into the BACT/ALERT^®^ VIRTUO^®^ instruments (hereafter referred to as “VIRTUO”) for incubation, platelet components are released as “culture negative to date”. If contamination is detected, all available components associated with the donation (red cells, plasma, cryoprecipitate, cryo-depleted plasma) are quarantined or recalled immediately.

On 21 March 2021, Lifeblood implemented an extension of platelet component shelf-life from five to seven days using large-volume delayed sampling (LVDS). The regulatory approval for safely extending the shelf-life of platelet components was supported by the demonstration of increasing the sensitivity of detecting low levels of bacterial contamination in platelet components, consequently improving recipient safety. Combining the LVDS protocol with a seven day platelet shelf-life had already been proven to be successful by other blood services [6,7,8].

This article describes the results from the routine BCS of platelet components in Australia and compares the 3.8-year period preceding VIRTUO implementation (1 January 2016 to 31 October 2019) to the 2-year period following VIRTUO and LVDS implementation (1 April 2021 to 31 March 2023). Data collected in the period November 2019 to March 2021 have been excluded from analysis as multiple changes were implemented nationally over this period.

## 2. Materials and Methods

**Figure 1:**
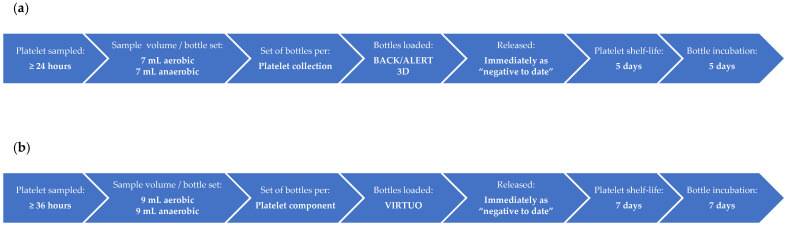
Bacterial contamination screening (BCS) protocols. (**a**) BCS protocol with platelet shelf-life of five days. (**b**) Large-volume delay sampling (LVDS) BCS protocol with platelet shelf-life of seven days.

BCS was introduced at Lifeblood in 2008 using the BACT/ALERT^®^ 3D automated microbial detection system (BioMérieux, Durham, NC, USA). The process was previously described by Borosak et al. and Thyer et al. [1,9]. There are currently four testing laboratories (Brisbane, Melbourne, Perth, and Sydney). Both buffy-coat-derived pooled platelet (pools of four) and apheresis platelet components are leucodepleted during manufacture and resuspended in platelet additive solution SSP+ (“PAS-E” manufactured by Macopharma, Mouvaux, France). Briefly (shown also in Figure 1a), buffy-coat-derived pooled platelet components were sampled 24 h post the phlebotomy of the “youngest” buffy coat in the platelet pool. Apheresis platelet components were sampled at a minimum time of 24 h post phlebotomy. Double and triple apheresis donations were pooled, sampled, and then heat-sealed into individual components. Samples of 7 mL each were inoculated into one aerobic and one anaerobic culture bottle, with double and triple apheresis collections only having one set of culture bottles. Following the loading of inoculated culture bottles into the incubator for continuous monitoring, the platelet components were released into inventory, available for immediate issue. Platelet shelf-life was five days from phlebotomy. Culture bottles were initially incubated for a period of seven days, but incubation was reduced to five days in September 2018.

Between 27 November 2019 and 3 February 2020, the VIRTUO instruments (BioMérieux, Durham, NC, USA) were implemented at all four testing sites. A staggered approach was deployed to institute the various changes in the testing process nationally. Firstly, testing sensitivity was increased by inoculating a larger sample volume (9 mL) into each culture bottle (completed in May 2020), followed by higher volume sampling for double and triple apheresis collections with two and three sets of culture bottles, respectively (completed in November 2020). Finally, the LVDS approach was implemented on 21 March 2021. Between 9 November and 8 December 2021, the VIRTUO software was upgraded (VIRTUO R3), which included a predictive loading feature to reduce false-positive results during the bulk loading of culture bottles or other temperature events.

With the implementation of LVDS (Figure 1b), buffy-coat-derived pooled platelet components were sampled 36 h post phlebotomy of the “youngest” buffy coat in the platelet pool and apheresis platelet components were sampled at a minimum time of 36 h post phlebotomy. Adequate testing volume was removed from each platelet donation to allow samples of 9 mL each into one aerobic and one anaerobic culture bottle. For double and triple apheresis platelet components, the component volume was distributed evenly between the packs prior to sampling. Following sampling, the packs were heat-sealed into individual components. There was one set of culture bottles for buffy-coat-derived pooled platelet components and single apheresis platelet components, two sets for double apheresis platelet components, and three sets for triple apheresis platelet components. Once inoculated culture bottles were loaded into the incubator, the platelet components were released into inventory with a shelf-life of seven days, available for immediate issue. Culture bottles were incubated for seven days.

Approximately 55% of whole blood donations collected annually are processed into pooled platelets. For collection sites where whole blood collection is not routinely used for pooled platelet manufacture or if the site does not manufacture apheresis platelets, 1% of red cells from that site are BCS-tested.

The actions following a bottle signalling positive are described in Thyer et al., with a final status assigned based on the Gram stain and culture results [1]. A “false-positive” status is assigned when the original and repeat samples are Gram-stain-negative and culture-negative, a “confirmed positive” (true positive) status for when the same organism is cultured in both the original and repeat samples, and an “indeterminate” status for when an organism is cultured in the original sample but cannot be confirmed due to unavailability of components for repeat testing, the repeat sample having a negative culture result, or the repeat sample having a positive culture result, but a different organism is identified.

Prior to introducing more than one set of culture bottles proportional to the type of apheresis platelet collection, the denominator used to calculate positive rates was “per collection”, that is, components from a double or triple apheresis collection were considered to be “one collection”. Although, currently, there are multiple sets of bottles with LVDS, a single positive bottle affects all components in the collection; therefore, the same denominator of “per collection” has been applied for comparative positive rates between periods.

The methods for data collection of BCS-positive components and adverse transfusion reactions were performed as outlined in Thyer et al. [1]. The number of platelet components manufactured and issued were obtained through internal Lifeblood reports.

Statistical analysis was performed using R version 4.2.1. Relative risks (RR) were calculated using unconditional maximum likelihood estimation with 95% confidence intervals (CI) derived by normal approximation (Wald). Chi-squared tests were used to obtain p-values. For time to detection, Kaplan–Meier survival curves were constructed, then compared using the log-rank test. Binomial exact CIs are presented for TTBI rates.

## 3. Results

### 3.1. Total Number of Platelet Donations Screened and the Rates for Initial Machine-Positive, False-Positive, Confirmed Positive, and Indeterminate

#### 3.1.1. Comparison of BACT/ALERT 3D and BACT/ALERT VIRTUO

For the period prior to LVDS (January 2016 to October 2019), with screening performed on the BACT/ALERT 3D (hereafter referred to as “pre-LVDS”), and for the period following LVDS (April 2021 to March 2023), with screening performed on the VIRTUO (hereafter referred to as “post LVDS”), the rates for initial machine-positive results and the assigned statuses for each are shown in Table 1 and Table 2, respectively.

Culture bottles in the pre-LVDS period were incubated for seven days from January 2016 to August 2018, then five days from September 2018 to October 2019.

The initial machine-positive results were 18% less likely to occur in the post LVDS period (RR 0.82, 95% CI 0.76–0.88, *p* < 0.001). This was attributable to a significant decrease in false-positive results, which were 41% less likely to occur in the post LVDS period (RR 0.59, 95% CI 0.52–0.68, *p* < 0.001); the rate of confirmed positive and indeterminate results remained stable (RR 0.995, 95% CI 0.91–1.09, *p* = 0.92).

A significant decrease in false-positives was observed for both platelet types (*p* < 0.001 for each), but a greater effect was observed for apheresis (RR 0.46, 95% CI 0.36–0.58) than pooled (RR 0.72, 95% CI 0.62–0.85) platelets.

#### 3.1.2. BACT/ALERT VIRTUO following R3 Software Upgrade

All testing sites completed the VIRTUO software upgrade (VIRTUO R3) by 8 December 2021. For the period December 2021 to March 2023, a total of 166,031 platelet donations were screened. The initial machine-positive rate was 0.32% (*n* = 529), comprising a false-positive rate of 0.07% (*n* = 110), a confirmed positive rate of 0.12% (*n* = 194), and an indeterminate rate of 0.14% (*n* = 225).

The software upgrade resulted in a pronounced decrease in false-positive results, which were 71% less likely to occur in this period compared to April 2021 to October 2021 (RR 0.29, 95% CI 0.23–0.37, *p* < 0.001). There was a corresponding decrease in initial machine-positives (RR 0.63, 95% CI 0.55–0.72, *p* < 0.001) and no effect on the rate of confirmed positive and indeterminate results (RR 0.92, 95% CI 0.77–1.08, *p* = 0.30).

### 3.2. Time to Detection in Culture Bottles with a Positive Gram Stain or Culture

**Figure 2:**
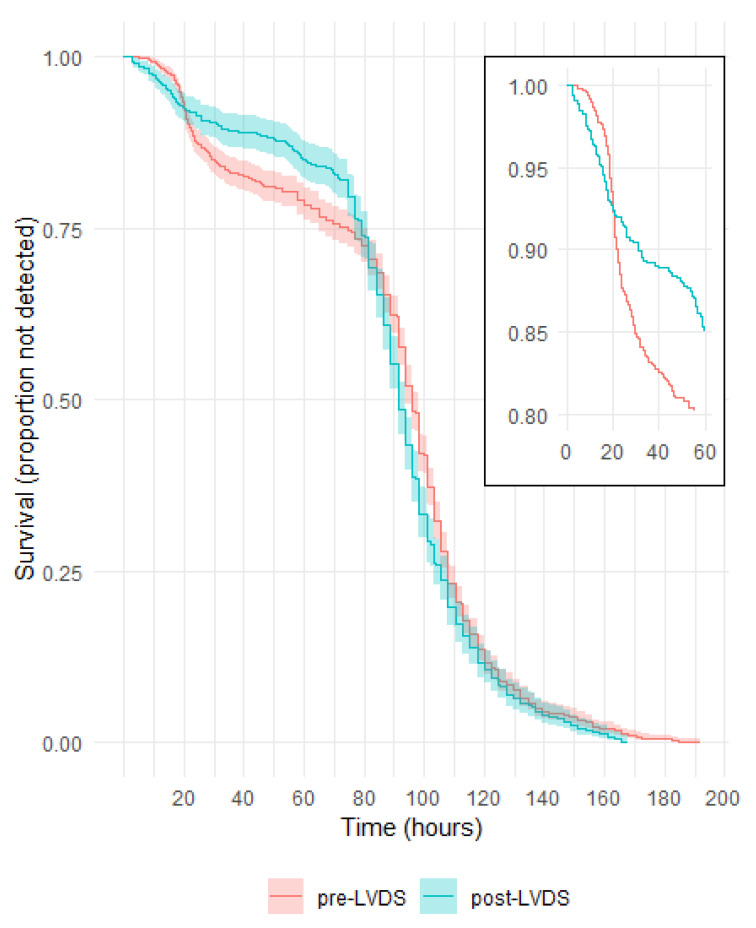
Survival curves for time to detection in culture bottles with a positive Gram stain or culture.

For culture bottles where bacteria were subsequently identified (i.e., a status of confirmed positive or indeterminate), the times to detection are plotted in Figure 2. Where multiple bottles from the same platelet collection flagged positive, the earliest time to detection was used.

The survival curves show a quicker detection of organisms within approximately the first 20 h post LVDS, followed by more detections pre-LVDS between approximately 20 and 80 h. The difference between the survival curves was significant (*p* = 0.01).

The majority of bottles flagged positive from 72 h of incubation (76% and 82% in the periods pre-LVDS and post LVDS, respectively, Figure S1).

### 3.3. Commonly Identified Organisms

The organisms identified pre-LVDS and post LVDS are shown in Table 3. Only the commonly identified organisms isolated from platelet collections are listed (with full lists provided in Table S1 and Table 4, respectively). The majority of the organisms identified were skin flora, with *Cutibacterium* spp. and coagulase-negative staphylococci accounting for more than 88% of all organisms identified.

### 3.4. Pathogenic Potential of Organisms Identified in Post LVDS Platelet Components and Their Time to Detection

Post LVDS implementation, 649 platelet collections had an identified organism. Organisms were classified according to pathogenic potential based on the likelihood of causing clinically significant disease. The number of isolates and the time to detection are shown in Table 4.

**Figure 3:**
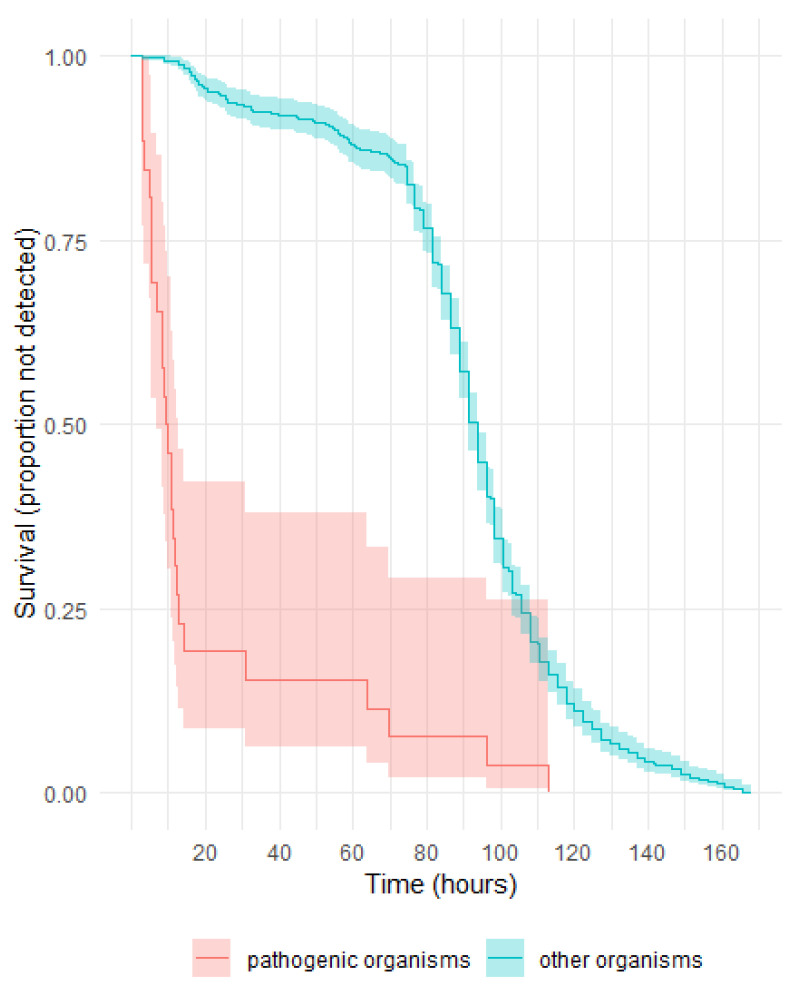
LVDS: survival curves for time to detection of pathogenic vs. other organisms.

As shown in Figure 3, more than 75% of pathogenic organisms were detected before 20 h; those detected after 24 h were rare slow-growing anaerobes. The majority of other organisms flagged positive from approximately 80 h, and of these, *Cutibacterium* spp. was the only organism isolated (Figure S2). The difference between the survival curves for pathogenic vs. other organisms was significant (*p* < 0.001).

**Table 4:** LVDS: number of organisms identified and the time to detection (time range in hours).

	Organism	<6	≥6–<12	≥12–<18	≥18–<24	≥24–<48	≥48–<72	≥72–<96	≥96–<120	≥120
Pathogenic										
Skin	*Staphylococcus aureus*		6	1						
	*Streptococcus dysgalactiae*	1								
	*Streptococcus pyogenes*	1								
	*Staphylococcus lugdunensis*			1						
Environment	*Bacillus cereus*	3	1							
Gut	*Bacteroides* spp.						2		2	
	*Escherichia coli*	1								
	*Enterococcus hirae*		1							
	*Enterococcus faecalis*		1							
	*Campylobacter fetus*					1				
Throat/lungs	*Streptococcus pneumoniae*		1							
Skin/urine	*Staphylococcus saprophyticus*			1						
Opportunistic										
Gut/environment	*Serratia marcescens*	2								
Low pathogenicity										
Skin	*Cutibacterium* spp.					1	5	246	197	74
	Coagulase negative staphylococci ^1^		1	12	4	13	29	3	2	1
	*Micrococcus* spp.					1	1	1		
	*Streptococcus sanguinis* ^2^				2					
	*Corynebacterium* spp.							1	1	
	*Lactococcus* spp.		1							
	*Kocuria* spp.				1					
	*Streptococcus cristatus*				1					
	*Dermacoccus nishinomiyaensis*								1	
	*Cutibacterium* spp./*Micrococcus* spp. ^3^								1	
	Unidentified Gram-positive bacilli								1	
Environment	*Bacillus* spp./*Paenibacillus* spp. ^4^	1		6	1	2	5	2		1
	*Pseudomonas oryzihabitans*						1			
Environment/skin	*Bacillus thuringiensis/Herbaspirillium huttiense/S. epidermidis* ^3^	1								
Skin/gut	*Streptococcus gallolyticus* ^2^		1							
Gut	*Eucobacterium callanderi*					1				

^1^ Excluding *S. lugdunensis* and *S. saprophyticus.* ^2^ May be associated with risk of underlying donor-related health conditions such as infective endocarditis or gastro-intestinal pathology. ^3^ Multiple organisms identified. ^4^ Excluding *B. cereus.*

#### Pathogenic Organisms and Their Time to Detection in Aerobic and Anaerobic Culture Bottles

A total of 26 pathogenic organisms were identified in 20 pooled platelet components and six apheresis platelet collections (Table 5). The organism was only identified from the anaerobic bottle in eight of these.

### 3.5. Frequency of Platelet Component Expiry: 5-Day vs. 7-Day Shelf-Life

In the period January 2016 to October 2019, when platelet shelf-life was five days, 571,897 platelet components were manufactured, with a combined total of 70,403 (12.3%) discarded by Lifeblood and hospitals due to inventory expiry. In the April 2021 to March 2023 period, after platelet shelf-life was extended to seven days with the use of LVDS, 318,814 platelet components were manufactured with 21,228 platelet components (6.7%; *p* value < 0.001) discarded due to expiry.

### 3.6. Incidence of TTBI Cases Reported to Lifeblood following the Implementation of BCS in April 2008

The five TTBI cases reported between 2008 and 2015, three associated with platelet components and two associated with red cell components, were described in Thyer et al. [1].

From 2016 to 2023, there were five confirmed or highly probable cases—four from platelet components and one from a red cell component. These are shown in Table 6. There have been no TTBI cases from October 2019 to June 2023.

Since the implementation of BCS in April 2008, there has been a total of seven TTBI cases associated with platelets and three cases associated with red cells. This equates to a rate of 0.34 (95% CI 0.14–0.71) per 100,000 platelet components issued, and a rate of 0.028 (95% CI 0.006–0.082) per 100,000 red cell components issued. There have been no cases of TTBI reported to Lifeblood since the implementation of LVDS.

## 4. Discussion

### 4.1. Principal Findings

Lifeblood introduced LVDS on the VIRTUO in conjunction with extending the platelet shelf-life to seven days just over two years ago. The data presented show this protocol is very safe, with no TTBI cases during this time, compared to the five confirmed or highly probable TTBI cases reported in the preceding time period from January 2016 to October 2019 (Table 6). All the bacterial species with high pathogenic potential grew within 24 h of inoculation, with the exception of rare fastidious or obligate anaerobes. The likelihood of these fastidious organisms surviving in components is negligible due to their growth requirements. The more rapid detection of pathogenic bacterial species when combined with the time to transport the platelet to the hospital allowed the impacted components, especially platelets, to be intercepted well before hospitals are likely to transfuse them. For this reason, Lifeblood does not require an extra hold period, such as the 6-12 h performed by other blood services [6,7,8]. In addition, the average age of a platelet component when issued by Lifeblood is four days (internal data), which also means platelet components which may be contaminated with pathogenic bacterial species are highly likely to still be within Lifeblood premises.

*Cutibacterium acnes*, which is the most common isolate from BCS, grows only after several days of incubation, and there is a clear delineation in the time periods between the pathogenic and non-pathogenic species. Comparing the commonly identified bacteria pre- and post LVDS, pathogens like *S. aureus*, *Streptococcus* spp., and *Bacteroides* spp. comprise a higher proportion of detections in the period after LVDS, whereas skin and environmental contaminants are predominant in the pre-LVDS period (Table 3).

Our data show that the VIRTUO has lower false-positive rates than other platforms, notably since the R3 software upgrade (Table 2). This has contributed to significantly decreased wastage from component discard, eliminated unnecessary clinician notification and recipient follow up, and resulted in an overall improvement in platelet inventory.

Components with positive culture results are detected more rapidly after implementing LVDS, with a higher proportion of positive cultures being detected within 20 h of incubation (Figure 2). This is likely to be a combination of increased testing sensitivity using the LVDS protocol and the faster time to detection expected with the VIRTUO [10]. The inoculation of both aerobic and anaerobic bottles for BCS is routine practice at Lifeblood, and the analysis of data showed that most pathogens are detected in both bottles with similar incubation time. However, obligate anaerobic bacteria like *Bacteroides* spp. and rare fastidious bacteria like *Campylobacter fetus* were detected in anaerobic bottles only (Table 5). This highlights the advantage of including both aerobic and anaerobic blood culture bottles for the isolation of microorganisms with a wide range of growth requirements, and possibly also to identify underlying donor health issues. While the use of the anaerobic culture bottle adds value by increasing detections (estimated to be 1 in 17,000 platelet components, excluding *Cutibacterium* spp.), it also increases overall costs through testing and platelet component wastage [11].

Although this analysis includes only 24 months of data post LVDS implementation, the trend is promising and seems to indicate improvement in the safety of the platelet supply.

### 4.2. Strengths and Weaknesses of the Study

This study continues the comprehensive assessment of BCS of platelets in Australia described in the earlier papers by Borosak et al. and Thyer et al. [1,9]. Lifeblood collects 1.6 million donations annually, from which approximately 160,000 platelet components are manufactured, giving a substantial sample size. The main limitation of the study is that there are now so few TTBI episodes reported to Lifeblood that it will take many more years to fully assess the impact of the new strategy on the incidence of TTBIs. It is noted that there were no cases of TTBI reported in any recipients who received platelet components that subsequently had a positive culture result, likely due to the majority of isolates being non-pathogenic nature (*C. acnes* and coagulase negative staphylococcus species).

### 4.3. Strengths and Weaknesses in Relation to Other Studies, Discussing Particularly any Differences in Results

Canadian Blood Services (CBS) reported on their LVDS results using the BACT/ALERT 3D system [7]. CBS’s rates were 0.41% for initial machine-positive (Lifeblood: 0.38%), 0.21% for false-positive (Lifeblood: 0.12%), 0.08% for confirmed positive (Lifeblood: 0.12%), and 0.11% for “indeterminate”, comprising the other categories where the initial result could not be confirmed (Lifeblood: 0.14%). Variations between the two processes (for example, CBS use three aerobic and one anaerobic culture bottles for double apheresis collections and the BACT/ALERT 3D) do not allow for a direct comparison; however, the rate of false-positives is lower at Lifeblood. This is consistent with what is expected from the VIRTUO. The overall positive culture rate appears higher at Lifeblood (0.26% vs. 0.20%), which may be due to the increased number of anaerobic culture bottles used for double and triple apheresis platelet collections.

In 2017, the United Kingdom National Health Service Blood and Transplant (NHSBT) also reported on their LVDS experience using the BACT/ALERT 3D system [6]. NHSBT’s rates were 0.37% for initial machine-positive, 0.19% for false-positive, 0.03% for confirmed positive, and 0.15% for indeterminate.

Lifeblood did not perform repeat testing of unused, initially screened platelet components beyond their seven day shelf-life to provide an indirect measure for a false-negative rate. CBS tested 5310 out-dated platelet concentrates, and subsequently categorised five as false-negative (approximately 1 in 1060) [7]. NHSBT and Héma-Québec re-tested 4515 and 4536 platelet components, respectively, and detected no positives [6,12].

In Australia, the estimated TTBI rate is 1 in 290,000 per platelet component issued. This is comparable to TTBI rates of approximately 1 in 244,000 and 1 in 300,000 from CBS and NHSBT, respectively [6,7].

### 4.4. Meaning of the Study: Possible Mechanisms and Implications for Clinicians or Policymakers

Our findings show that the combination of LVDS and VIRTUO allows the earlier detection of pathogenic bacterial species. This could potentially reduce the likelihood of TTBI. The extra two days of shelf-life, along with reduced wastage due to decrease in false-positivity rates, has considerably increased platelet inventory levels, which is particularly important in the post-coronavirus disease 2019 era, as there has been ongoing difficulties recruiting sufficient numbers of donors to maintain adequate inventory levels.

Our data suggest all clinically significant pathogenic bacteria grew within 72 h of incubation and there is little added value in extending the culture beyond 72 h using LVDS. Hence, Lifeblood intends to submit an initial proposal to the regulator to reduce the incubation period from seven days to five days, as there appears to be little gain by identifying the *Cutibacterium* spp. growing on days six and seven, which are of no clinical consequence to the recipients. A review of the literature shows that there are no convincing episodes of TTBI caused by *C. acnes*. Historic papers postulating its role as a possible cause of transfusion-related sepsis failed to grow the organism in patients’ blood cultures, casting doubt on its pathogenic role in those circumstances [13,14]. There is a claim that *C. acnes* can cause chronic infections in relevant clinical settings [15]; however, there is no evidence that *C. acnes* growth from platelets is the source of infection; rather there are multiple potential sources that can cause infections, especially sources associated with implanted devices, such as recipients’ own normal flora, surgery and prostheses. Therefore, the identification of *Cutibacterium* spp. has no demonstrated impact on the safety of the blood supply, as there are no reported cases of TTBI attributed to this organism and there is no advantage by extending the culture to identify these, resulting in unnecessary component recalls, component wastage, and untoward anxiety for recipients, donors, and treating clinicians.

The risk of TTBI from platelet transfusion could be mitigated further through pathogen reduction technology (PRT), which has been introduced by many blood services, or implementing a very sensitive BCS system. Following a recent internal cost–benefit analysis, PRT has not been introduced in Australia. Earnshaw et al. compared LVDS and PRT for platelets, and found LVDS was associated with lower costs, higher platelet component availability, and lower platelet component use, with similar levels of adverse events [16].

### 4.5. Unanswered Questions and Future Research

As TTBI has become uncommon, in a similar way to that in which transmissions of viruses such as human immunodeficiency virus, hepatitis B virus, and hepatitis C virus are exceedingly rare, we consider that the modelling of residual risk should now be carried out for the bacterial contamination of components in the same way it is for the viruses [17], so that a calculated estimate can be used to judge the worth of future methods to reduce the incidence of TTBI.

## 5. Conclusions

LVDS using the VIRTUO in conjunction with increasing the platelet shelf-life is safe. Pathogenic bacteria are detected more rapidly. No TTBI episodes have been reported to Lifeblood during the time of the program. As TTBIs have become rare, it might be necessary to undertake modelling to estimate the future risk of this infection.

## Figures and Tables

**Table 1:** Percentages and numbers of platelet collections screened prior to LVDS on BACT/ALERT 3D and the assigned status: cumulative totals (January 2016–October 2019) *.

Platelet Collection	Total Number Screened	Initial Machine-Positive	False-Positive	Confirmed Positive	Indeterminate
Pooled	362,051	0.45 (1627)	0.15 (549)	0.13 (461)	0.17 (617)
Apheresis	107,588	0.52 (558)	0.38 (407)	0.05 (52)	0.09 (99)
Total	469,639	0.47 (2185)	0.20 (956)	0.11 (513)	0.15 (716)

* Data are reported as percentage (number).

**Table 2:** Percentages and numbers of platelet collections screened with LVDS on VIRTUO and the assigned status: cumulative totals (April 2021–March 2023) *.

Platelet Collection	Total Number Screened	Initial Machine-Positive	False-Positive	Confirmed Positive	Indeterminate
Pooled	204,811	0.38 (783)	0.11 (225)	0.14 (279)	0.14 (279)
Apheresis	44,147	0.38 (167)	0.17 (76)	0.05 (24)	0.15 (67)
Total	248,958	0.38 (950)	0.12 (301)	0.12 (303)	0.14 (346)

* Data are reported as percentage (number).

**Table 3:** Percentages for commonly identified organisms in platelet collections.

Pre-LVDS		Post LVDS	
*Cutibacterium* spp. including *C. acnes*	72.1	*Cutibacterium* spp. including *C. acnes*	80.7
Coagulase-negative staphylococci	15.9	Coagulase-negative staphylococci	10.5
*Micrococcus* spp.	3.0	*Bacillus* spp./*Paenibacillus* spp.	3.5
*Bacillus* spp./*Paenibacillus* spp.	2.4	*Staphylococcus aureus*	1.1
*Streptococcus* spp.	1.6	*Streptococcus* spp.	1.1
*Corynebacterium* spp.	1.4	*Bacteroides* spp.	0.6
*Kocuria* spp.	0.9	*Micrococcus* spp.	0.6

**Table 5:** LVDS: time to detection (hours) in aerobic and anaerobic culture bottles for pathogenic organisms.

Organism	Platelet Collection	Aerobic Culture Bottle			Anaerobic Culture Bottle		
		1	2	3	1	2	3
*S. aureus*	Apheresis double	6.6	ND	-	7.2	ND	-
		8.6	ND	-	9.1	9.2	-
		11.3	ND	-	11.7	ND	-
	Pooled	9.4	-	-	8.4	-	-
		10.6	-	-	11.4	-	-
		10.8	-	-	11.5	-	-
		ND	-	-	12.1	-	-
*B. cereus*	Pooled	3.0	-	-	2.8	-	-
		3.2	-	-	3.2	-	-
		5.2	-	-	5.5	-	-
		ND	-	-	9.7	-	-
*Bacteroides* spp.	Apheresis double	ND	ND	-	69.6	ND	-
	Pooled	ND	-	-	63.7	-	-
		ND	-	-	96.0	-	-
		ND	-	-	112.8	-	-
*S. marcescens*	Pooled	2.8	-	-	2.8	-	-
		5.4	-	-	5.9	-	-
*E. coli*	Apheresis triple	3.4	3.4	3.4	2.7	3.0	3.5
*S. pyogenes*	Pooled	5.9	-	-	5.2	-	-
*S. dysgalactiae*	Pooled	6.0	-	-	4.7	-	-
*S. saprophyticus*	Apheresis double	12.9	ND	-	20.6	ND	-
*S. lugdunensis*	Pooled	14.9	-	-	14.0	-	-
*C. fetus*	Pooled	ND	-	-	35.7	-	-
*E. hirae*	Pooled	8.8	-	-	8.1	-	-
*E. faecalis*	Pooled	9.7	-	-	9.4	-	-
*S. pneumoniae*	Pooled	ND	-	-	11.7	-	-

“ND” = not detected, “-“ = no culture bottle.

**Table 6:** Organisms, component type, and outcome in confirmed or highly probable transfusion-transmitted bacterial infection cases in Australia, 2016–2022.

Calendar Year	Count	Implicated Organism	Component	Recipient Outcome	BCS Result
2016	2	*S. aureus*	Double apheresis platelet	Major morbidity in both recipients	Negative
2017	0	None			
2018	1	*Yersinia enterocolitica*	Red cell	Recovered	Negative ^1^
2019	2	*S. aureus* Group G *Streptococcus*	Triple apheresis platelet Pooled platelet	Recovered Death	Negative
					Negative
2020	0	None			
2021	0	None			
2022	0	None			
2023	0 ^2^	None			

^1^ Associated pooled platelet. ^2^ To end of June.

## Data Availability

The data presented in this study are available on request from the corresponding author.

## Supplementary Materials

The following supporting information can be downloaded at: https://www.mdpi.com/article/10.3390/microorganisms11092346/s1, Figure S1: Percentages for time to detection (time ranges in hours) in culture bottles with a positive Gram stain or culture; Table S1: Pre-LVDS: number of organisms identified and the time to detection (time range in hours); Figure S2: LVDS: percentages for the types of organisms identified and the time to detection (time range in hours).
