# Supplementary material for: Safety Analysis of Extended Platelet Shelf-Life with Large-Volume Delayed Sampling on BACT/ALERT® VIRTUO® in Australia
Source: Microorganisms. 2023 Sep 19;11(9):2346. doi: 10.3390/microorganisms11092346 (PMC10535894; doi:10.3390/microorganisms11092346)

microorganisms

MDPI

Supplementary Materials

Figure S1: Percentages for time to detection (time ranges in hours) in culture bottles with a positive Gram stain or culture.

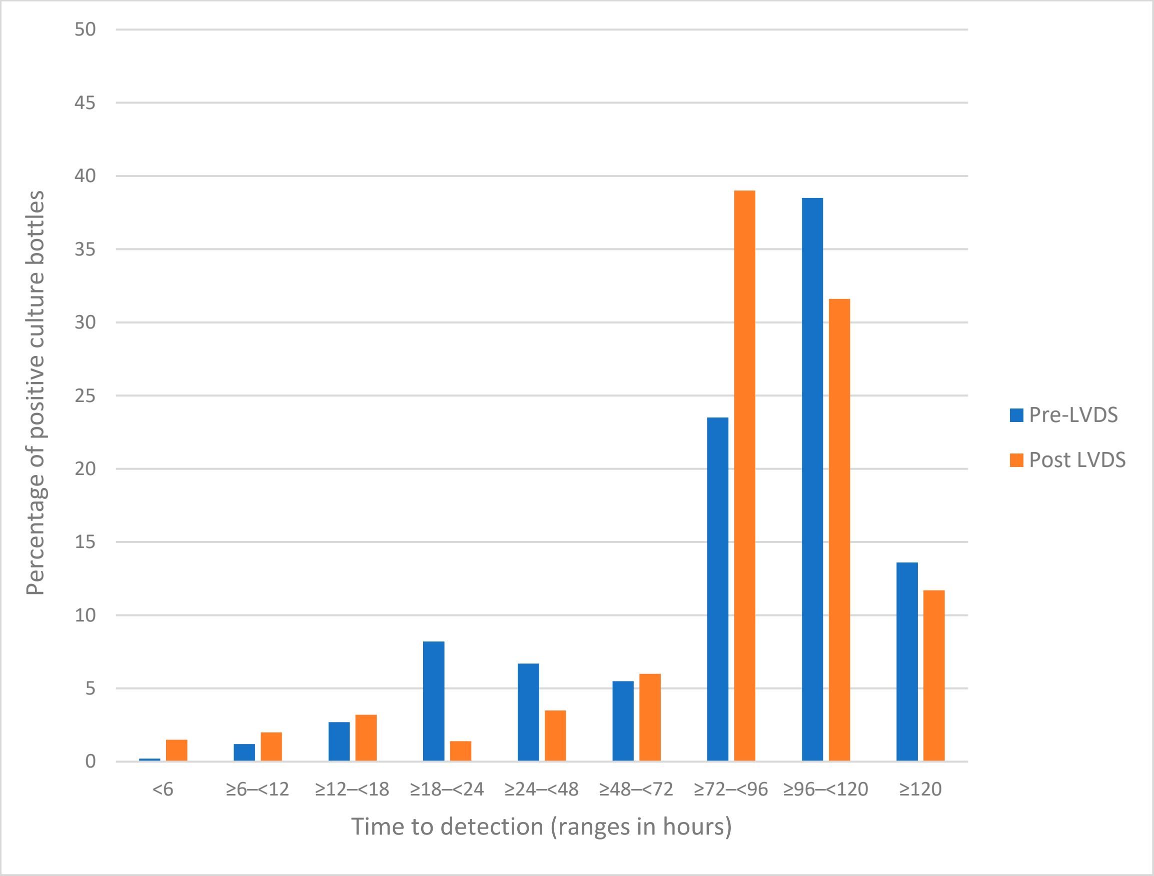

Table S1. Pre-LVDS: number of organisms identified and the time to detection (time range in hours).

	**Organism**	**<6**	**≥6–<12**	**≥12–<18**	**≥18–<24**	**≥24–<48**	**≥48–<72**	**≥72–<96**	**≥96–<120**	**≥120**
Pathogenic										
Environment	*Bacillus cereus*	1		4						
Skin	*Staphylococcus aureus*			2	1					
	Staphylococcus lugdunensis				2	1				
	Streptococcus dysgalactiae		2							
	Streptococcus pyogenes		1							
	S. dysgalactiae/*S. pyogenes* [1]		1							
	Group C streptococcus		1							
	S. aureus/Coagulase negative staphylococci [1,2]				1					
Skin/urine	*Staphylococcus saprophyticus*				1					
Throat/lungs	*Streptococcus pneumoniae*			5						
Throat/lungs/gut	*Klebsiella pneumoniae*		1							
Gut	*Streptococcus agalactiae*		2							
	Escherichia coli		1							
	Enterococcus faecalis			3						
	Bacteroides spp.				1					1
	Fusobacterium spp.					2				
Opportunistic										

Oral	*Streptococcus parasanguinis*				1	2				
	Streptococcus mitis			1					1	
	Streptococcus oralis				1					
	Gemella haemolysans				1					
	Parvimonas micra							1		
Urine	*Aerococcus viridans*				1					
Gut	*Finegoldia magna*					1				
Gut/environment	*Serratia marcescens*	1	3	1						
	Clostridium perfringens		1							
Low pathogenicity										
Skin	*Cutibacterium* spp.		1			2	5	269	451	152
	Coagulase negative staphylococci [2]			13	81	38	43	2	3	5
	Micrococcus spp.					17	9	3	5	1
	Corynebacterium spp.					1	1	7	3	4
	Kocuria spp.					7	4			
	Cutibacterium spp./Coagulase negative staphylococci [1,2]				2			2	1	
	Lactococcus spp.			1				1		
	Dermacoccus barathri								1	1
	Unidentified Gram-positive cocci					1	1			
	Micrococcus sp./Corynebacterium spp. [1]						1			
	Streptococcus sanguinis [3]/Coagulase negative staphylococci [1,2]				1					
Environment	*Bacillus* spp./*Paenibacillus* spp. [4]			3	6	9		2	3	2
	Collinsella aerofaciens					1			1	
	Dietzia spp.						1	1		
	Paecilomyces spp.								1	1
	Pseudomonas stutzeri				1					
	Unidentified Gram-positive bacilli						1			
	Virgibacillus proomii							1		
	Microbacterium spp.								1	
	Brevibacterium spp.									1
Skin/gut	*Streptococcus gallolyticus* [3]		1							
Skin/environment	*Micrococcus* spp./*Brevibacterium* spp. [1]						1			
	Cutibacterium spp./*Pseudomonas aeruginosa* [1]								1	

[1] Multiple organisms identified. [2] Excluding *S. lugdunensis* and *S. saprophyticus*. [3] May be associated with risk of underlying donor related health conditions such as infective endocarditis or gastro-intestinal pathology. [4] Excluding *B. cereus*.

Figure S2: LVDS: percentages for the types of organisms identified and the time to detection (time range in hours).

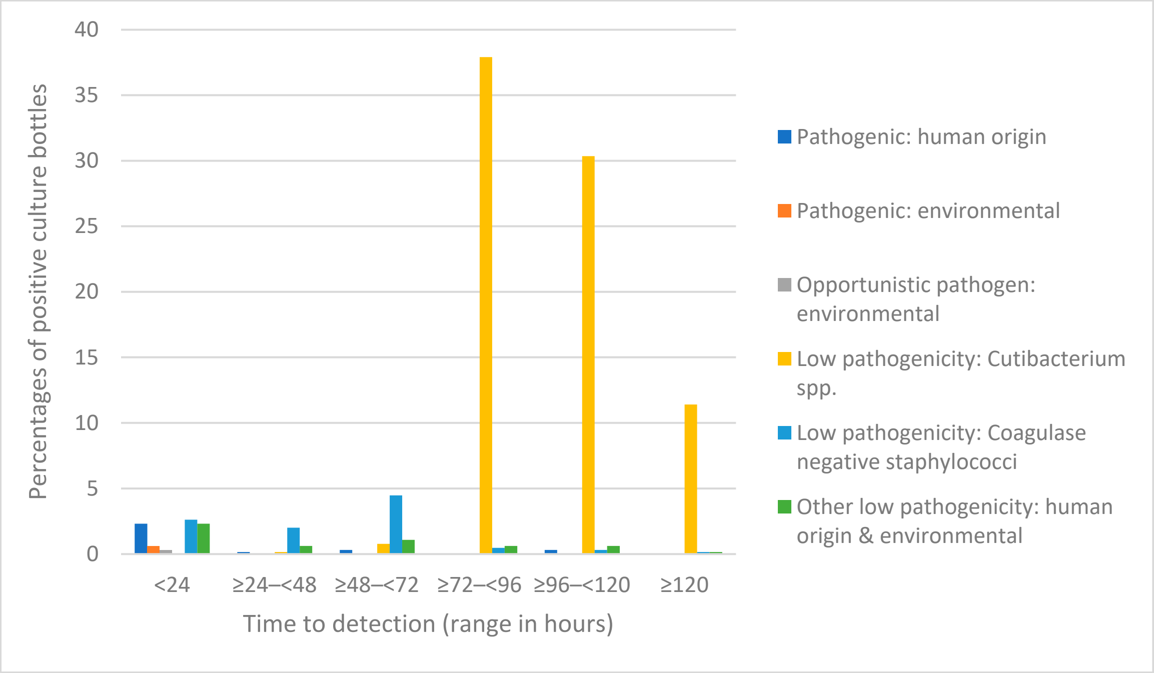

Supplement: Supplementary file 1 [file microorganisms-11-02346-s001.zip › microorganisms-2598513-supplementary.pdf]
